# Surface ligand chemistry on quaternary Ag(In_*x*_Ga_1−*x*_)S_2_ semiconductor quantum dots for improving photoluminescence properties†

**DOI:** 10.1039/d1na00684c

**Published:** 2021-12-10

**Authors:** Watcharaporn Hoisang, Taro Uematsu, Tsukasa Torimoto, Susumu Kuwabata

**Affiliations:** Department of Applied Chemistry, Graduate School of Engineering, Osaka University 2-1 Yamada-oka Suita Osaka 565-0871 Japan t-uematsu@chem.eng.osaka-u.ac.jp kuwabata@chem.eng.osaka-u.ac.jp; Innovative Catalysis Science Division, Institute for Open and Transdisciplinary Research Initiatives (ICS-OTRI), Osaka University 2-1 Yamada-oka Suita Osaka 565-0871 Japan; Department of Materials Chemistry, Graduate School of Engineering, Nagoya University Chikusa-ku Nagoya 464-8603 Japan

## Abstract

Ternary and quaternary semiconductor quantum dots (QDs) are candidates for cadmium-free alternatives. Among these, semiconductors containing elements from groups 11, 13, and 16 (*i.e.*, I–III–VI_2_) are attracting increasing attention since they are direct semiconductors whose bandgap energies in the bulk state are tunable between visible and near infrared. The quaternary system of alloys consisting of silver indium sulfide (AgInS_2_; bandgap energy: *E*_g_ = 1.8 eV) and silver gallium sulfide (AgGaS_2_; *E*_g_ = 2.4 eV) (*i.e.*, Ag[In_*x*_Ga_1−*x*_]S_2_ (AIGS)) enables bandgap tuning over a wide range of visible light. However, the photoluminescence (PL) quantum yield (10–20%) of AIGS QDs is significantly lower than that of AgInS_2_ (60–70%). The present study investigates how to improve the PL quantum yield of AIGS QDs *via* surface ligand engineering. Firstly, the use of a mixture of oleic acid and oleylamine, instead of only oleylamine, as the solvent for the QD synthesis was attempted, and a threefold improvement of the PL quantum yield was achieved. Subsequently, a post-synthetic ligand exchange was performed. Although the addition of alkylphosphine, which is known as an L-type ligand, improved the PL efficiency only by 20%, the use of metal halides, which are categorized as Z-type ligands, demonstrated a twofold to threefold improvement of the PL quantum yield, with the highest value reaching 73.4%. The same procedure was applied to the band-edge emitting core/shell-like QDs that were synthesized in one batch based on our previous findings. While the as-prepared core/shell-like QDs exhibited a PL quantum yield of only 9%, the PL quantum yield increased to 49.5% after treatment with metal halides.

## Abstract 2

The photoluminescence properties of Ag(In_*x*_Ga_1−*x*_)S_2_ quaternary semiconductor quantum dots are improved by ligand exchange with L-, X-, and Z-type ligands. Analyses of both the quantum dots and byproducts are performed for understanding the mechanism.

## Graphical abstract

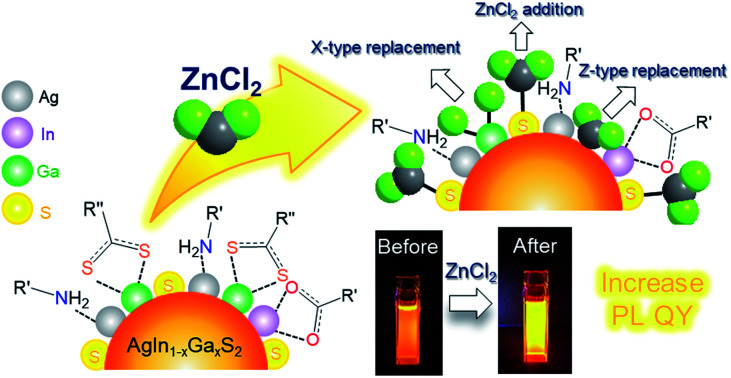

## Introduction

Semiconductor quantum dots (QDs) are photoluminescent materials that exhibit a high absorption coefficient and a monochromatic emission; these emissions range from visible to near infrared light. Recently, I_2_–VI, III–V and I–III–VI_2_ semiconductors have emerged as candidate materials for cadmium-free QDs,^1–3^ and they are usable in a variety of applications, including photovoltaics,^4–6^ light-emitting devices,^1,7–10^ and bio-imaging.^3,11–13^ The emission color of these QDs can be engineered by tuning their bandgap energy (*E*_g_) *via* their size; this is one of the unique characteristics of QDs, which is referred to as the quantum size effect.^14–16^ Furthermore, the bandgaps of I–III–VI_2_ semiconductors, a group that includes silver indium sulfide (AgInS_2_), copper indium sulfide (CuInS_2_), and their selenide derivatives, can also be controlled by adjusting their chemical composition and creating alloys from these materials. The degree of compositional freedom of ternary semiconductors seems to be higher than that available when using their binary counterparts, as demonstrated by the many reports on bandgap tuning *via* alterations of the chemical composition.^8,17–19^ Conversely, since the crystal structure of ternary I–III–VI_2_ semiconductors tolerates an off-stoichiometry ratio to a higher degree than their binary counterparts, the nanoparticles contain a higher concentration of defects in the crystal structures.^20^ In addition, the presence of more than three elements on the surface of the crystal facilitates the generation of various types of surface trap states. These defect levels often generate a spectrally broad emission and impair the monochromatic nature of QD fluorophores; considerable research efforts have been devoted to improving this property.

Our research groups have successfully generated a yellow band-edge photoluminescence (PL) from AgInS_2_ QDs using a coating of gallium sulfide (GaS_*y*_).^21^ The wavelength of the band-edge emission was further tuned by alloying AgInS_2_ and AgGaS_2_ (Ag[In_*x*_Ga_1−*x*_]S_2_, (AIGS)), whose bandgap energies are 1.8 and 2.4 eV, respectively.^22^ While uniformly incorporating less-reactive gallium was a substantial challenge, we found that gallium tris(*N*,*N*′-diethyldithiocarbamate) (Ga[DDTC]_3_) works well owing to its high reactivity in the production of metal sulfides, resulting in the successful synthesis of AIGS QDs at lower temperature; this process involved nearby ions (Ag^+^ and In^3+^) that were mixed in the reaction solution in the form of acetates.^23^ AIGS QDs with different In/Ga ratios were obtained with a well-controlled composition and crystal structure by changing the amount of Ga(DDTC)_3_, which acts as the source of both gallium and sulfur. However, the PL QY of the AIGS QDs decreased significantly upon increasing the amount of Ga(DDTC)_3_, which is due to the electron-accepting nature of the decomposition products of the DDTC ligands that are presumably bound on the surface. Since dithiocarboxylate complexes are efficient metal sulfide sources that generate a variety of sulfide semiconductors,^24,25^ a better understanding of these key complexes is strongly needed for further enhancements of the PL performance of the prepared QDs.

As anticipated from the PL quenching due to the adsorption of some types of ligands, the capping ligands provide a critical role in the passivation of QDs and the elimination of electronic trap states, along with their fundamental function, which is to determine the size and shape of the QDs.^26^ Therefore, ligand engineering must be one of the principal strategies in the enhancement of PL efficiency.^27–29^ To date, oleylamine (OLA), oleic acid (OA), and tri-*n*-octyl phosphine (TOP) have been widely employed as the ligands (and also as the solvents) in the synthesis of colloidal QDs.^30,31^ OLA provides a weak reducing effect, which assists the formation of metal and metal chalcogenide nanoparticles, whereas OA (when deprotonated) provides a stronger coordinating force to metal sites, resulting in a better passivation of nanoparticles. Both types of ligands have the ability to remove surface trap states and enhance the PL intensity of the QDs,^32,33^ and they have occasionally been utilized as a ligand (solvent) mixture with the aim of combining their properties.^34,35^

Surface ligands can be classified into L-, X-, and Z-types according to the number of electrons that the ligand provides in its neutral state.^36^ OLA is a typical L-type ligand, whereas OA presents features of both the L- and X-types and occasionally takes η^2^ coordination providing a stronger binding to the QD surface. Z-type ligands have a slightly different definition when they are part of the capping ligands of QDs; they include the Lewis-acidic metal complexes, such as metal oleate and halides. The Z-type ligand binds to electron-donating atoms, such as sulfur and selenium, which are occasionally dangling on the nanoparticles with unsaturated electron states. Recently, a significant increase in the PL intensity has been reported for II–VI and III–V semiconductor QDs, such as cadmium selenide (CdSe), cadmium telluride (CdTe), and indium phosphide (InP), which was achieved *via* post-treatment with Z-type ligands (metal halides).^28,37^ Since the surface-dangling chalcogenides that have two unbound electrons serve as hole trap states, their removal was effective in increasing the PL intensity, as demonstrated by the density functional theory calculations using a CdSe model nanocrystal (Cd_68_Se_55_).^27^ The effect of the ligands on the electronic structure of ternary and quaternary QDs has continually been investigated using L- and X-type ligands, and these studies have revealed the increased complexity of the surface structures when compared with those of binary semiconductors.^38,39^

In this work, we attempt to understand the types of ligands that can specifically passivate the dangling atoms or orbitals of the I–III–VI_2_ multinary QDs. Firstly, we study the influence of coordinating solvents on the synthesis of quaternary AIGS QDs using either OLA as an L-type ligand or a mixture of OLA and OA as L- and X-type ligands. Increasing the proportion of OA, which generally provides a stronger coordination than does OLA, made the size of the prepared QDs significantly smaller. Consequently, the PL wavelengths of the AIGS core QDs were red-shifted from 580 nm (OLA only) to 730 nm (OLA : OA = 1 : 2), and their PL QY increased by a factor of more than three. Since the ligands were found to have a substantial effect on the emission of the AIGS QDs, we subsequently investigated the effects of Z-type ligands (metal halides and carboxylate) on the PL of the AIGS QDs. A substantial improvement in the PL QY was achieved *via* post-treatment with zinc chloride (ZnCl_2_), one of the strong Lewis acids, which binds specifically to the electron-donating sites of the QDs. Furthermore, the same ZnCl_2_ treatment was applied to the band-edge emitting AIGS/GaS_*y*_ core/shell-like QDs that were prepared in a one-batch reaction from the raw material mixture. Although the original PL QY value was lower than that of the conventional GaS_*y*_-shelled AIGS QDs, a significant improvement in the PL QY was observed after the ZnCl_2_ treatment. The mechanisms of the PL QY improvement are discussed in relation to the addition and exchange of the ligands.

## Experimental section

### Chemicals

All anhydrous chemicals were stored in a nitrogen-filled glovebox. Indium acetate (In[OAc]_3_, anhydrous, 99.9%) and zinc oxide (ZnO, 99.9%) were purchased from Sigma-Aldrich. Indium chloride (InCl_3_, anhydrous, 2N), gallium chloride (GaCl_3_, 5N), and gallium nitrate (Ga[NO_3_]_3_·*n*H_2_O) were purchased from Mitsuwa Chemical Co., Ltd. OA (85%), tri-*n*-butyl phosphine (TBP, >95%), TOP (>85%), and zinc chloride (ZnCl_2_, 98.0%) were obtained from Tokyo Chemical Industry. Silver acetate (Ag[OAc], anhydrous, 99.9%), sodium *N*,*N*′-diethyldithiocarbamate (NaDDTC, 92.0%), hexane (anhydrous, 96%), and toluene (anhydrous, 99.8%) were purchased from Fujifilm Wako Pure Chemical. All chemicals were used without further purification. OLA was purchased from Fujifilm Wako Pure Chemical and was purified by vacuum distillation in the presence of calcium hydride.

### Synthesis of the AIGS QDs and AIGS/GaS_*y*_ core/shell-like QDs

The synthesis of the AIGS QDs was carried out following a recent report, which we modified.^23^ Ag(OAc) (0.2 mmol), In(OAc)_3_ (0.15 mmol), and Ga(DDTC)_3_ (0.4 mmol) were mixed with either 10 mL of OLA or 10 mL of the OLA : OA mixture solvent (2 : 1 or 1 : 2) in a two-necked round bottom flask. The solution was heated at 100 °C under vacuum to remove water and oxygen; thereafter, the atmosphere was changed to Ar gas. The solution temperature was increased to 150 °C at a rate of 5 °C min^−1^ and was maintained for 30 min to allow the AIGS QDs to grow. After cooling to room temperature, the final products were centrifuged for removing large precipitates. The QDs in the supernatant were precipitated through the addition of excess methanol, followed by dispersion in hexane. The QD solutions were then characterized and underwent post-treatment as described in the next section.

The synthesis of the AIGS/GaS_*y*_ core/shell-like QDs was performed at a temperature (280 °C) higher than that of the AIGS core (150 °C) using the same type and same amount of chemicals. It should be noted that a temperature ramp of 5 °C min^−1^ was applied to allow the core QDs to grow, and the highest temperature was maintained for 30 min. The purified core/shell-like QDs were subsequently dispersed in toluene for characterization and treatment, since the core/shell-like QDs had a lower solubility in hexane, which was used for the post-treatment of the core QDs.

### Post-treatment with Z-type ligands

A metal halide (MX_*n*_: *e.g.*, ZnCl_2_, InCl_3_, and GaCl_3_) stock solution was prepared using a modified version of a method from the literature.^29^ The MX_*n*_ (2 mmol) was dissolved in a mixture of hexane (10 mL) and OLA (1.3 mL) under vigorous stirring and stored at room temperature.^29^ For the treatment of the core/shell-like QDs, hexane was replaced by the same volume of toluene. Subsequently, the QD solution (20 nmol in terms of the particles) was added to 0.5 mL of the MX_*n*_ stock solution (molar ratio: MX_*n*_ : QD = 5000 : 1); thereafter, the total volume of the solution was adjusted to 3 mL through dilution with hexane or toluene. The mixture solution was vigorously stirred for 24 h under ambient conditions. Subsequently, the solution was centrifuged to remove unbound or unreacted ligands, and the treated QDs were then purified by adding a poor solvent. Finally, the purified QDs were dispersed in hexane for optical measurements. The molar ratio of MX_*n*_ to the QDs was varied between 1000 and 15 000.

### Post-treatment with L-type ligands

The desired amount of TBP or TOP (5000 ligands/QD) was added to the QD solution, and the solution was diluted with chloroform. The mixture solution was stirred under ambient condition and incubated for 24 h (a note of caution: TBP can be flammable if a small spill is wiped up with a Kimwipe).

### Material characterization

Ultraviolet-visible (UV-vis) absorption and PL spectra were obtained using a JASCO V-670 UV-vis spectrophotometer and a JASCO FP-8600 spectrofluorometer, respectively. PL QY measurements were carried out using a Hamamatsu PMA12 spectrofluorometer equipped with an integrating sphere. PL lifetimes were measured with a Hamamatsu Quantaurus-Tau C11367 compact fluorescence lifetime instrument using a pulsed light-emitting diode source at room temperature (380–1030 nm). Proton nuclear magnetic resonance (^1^H NMR) spectroscopy was conducted using a JEOL 400 MHz NMR spectrometer. The functional group on the QD surface was characterized using a JASCO FT/IR-6200 Fourier-transform infrared (FT-IR) spectrometer. The morphology of the QDs was observed *via* a Hitachi H-7650 transmission electron microscopy (TEM) instrument at an acceleration voltage of 100 kV. Powder X-ray diffraction (XRD) measurement was performed using a Rigaku SmartLab X-ray diffractometer equipped with a parallel beam/parallel slit analyzer. The chemical composition of the QDs was determined using a Hitachi S-3400N scanning electron microscopy instrument equipped with an AMETEK EDAX X1 energy-dispersive X-ray detector. The surface chemical electronic state and composition of the QDs were measured using a Shimadzu KRATOS AXIS-165x X-ray photoelectron spectroscopy (XPS) instrument equipped with an aluminum target.

## Results and discussion

### Surface ligands on the AIGS QDs

**Fig. 1:**
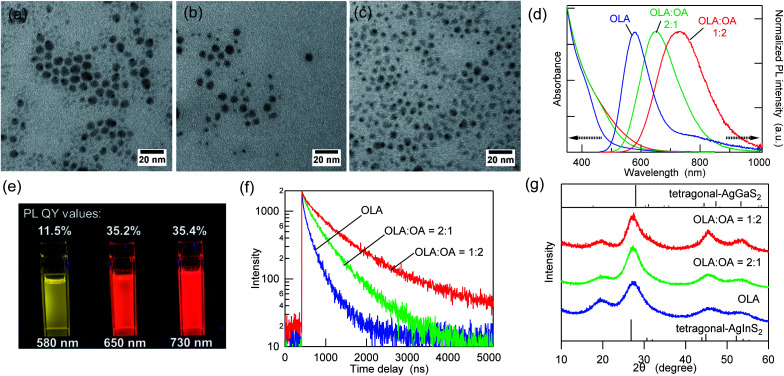
TEM images of the AIGS QDs synthesized using (a) OLA, (b) OLA : OA = 2 : 1, and (c) OLA : OA = 1 : 2. (d) Comparison of the UV-vis absorption and PL spectra of the as-synthesized AIGS QDs with different ligands (OLA, OLA : OA = 2 : 1, and OLA : OA = 1 : 2). (e) Photographs of the as-synthesized AIGS QDs under UV radiation (365 nm) with the label giving the corresponding PL QY value and PL emission wavelength; from left to right, the photos are of the samples with OLA, OLA : OA = 2 : 1, and OLA : OA = 1 : 2, and (f) PL decay curves at each emission wavelength. (g) XRD patterns of the as-synthesized AIGS QDs and reference XRD patterns of tetragonal-AgInS_2_ (ICSD 077-6632) and tetragonal-AgGaS_2_ (ICDD 027-0615).

To investigate the effect of the coordinating solvents, the AIGS QDs were synthesized either in pure OLA or in a mixture of OLA and OA (2 : 1 or 1 : 2). The use of OA alone as a solvent is inappropriate because amine is required for the formation of metal sulfide from metal dithiocarbamate complexes.^40,41^Fig. 1a–c and ESI Fig. S1† show the TEM images and size distribution of the as-synthesized AIGS QDs. The AIGS QDs synthesized with pure OLA were the largest (6.2 ± 1.0 nm), whereas they became slightly smaller (5.9 ± 1.7 nm) when the OLA : OA = 2 : 1 mixture solvent was used. Additionally, the particles became apparently small (4.6 ± 0.6 nm) when synthesized in OLA : OA = 1 : 2. These results agree with previous findings, according to which, when OA is deprotonated by OLA, it has a higher binding energy to the QDs than OLA has, since multiple binding modes (bidentate and bridged) are allowed.^42^ Therefore, the use of the OLA/OA mixture solvent resulted in smaller QDs due to their growth suppression.

To verify the effect of OA, the surface ligands of the resulting QDs were characterized *via* FT-IR and ^1^H NMR spectra. The obtained results are shown in Fig. S2.† In the FT-IR spectra, the C=O stretching band of the metal-coordinated carboxylate groups (1580 cm^−1^) became stronger as the OA proportion increased, whereas the small peak was found at the same position in the pure OLA sample owing to its spectral overlap with the C–H bending modes (Fig. S2a†). Conversely, the N–H stretching band (3200 cm^−1^) became weaker with an increase in the OA proportion. In the ^1^H NMR spectra, the identification of the two types of ligands proved impossible since the methylene peaks located next to and second closest to the functional groups (no. 2 and 4 in the figure, respectively) were broadened considerably and became almost invisible (Fig. S2b†). However, the broadening of the peaks, not only of the two aforementioned methylene peaks but also of the other observable peaks, for the AIGS QD sample ensured that all ligands were bound on the particles, leaving no free ligands after purification. Since the samples for the IR spectra were purified in the same manner, the signals observed in Fig. S2a† originated from the surface ligands.

Fig. 1d shows the intensity-normalized UV-vis absorption and PL spectra of the three AIGS QDs synthesized in different solvents. The absorption edge wavelengths of the three AIGS QDs were almost identical, whereas the QDs prepared in pure OLA showed a weaker absorption between 500 and 600 nm. By comparison, the apparent red shift of the PL peak positions and the threefold increase in the PL QY value were observed upon increasing the OA proportion (Fig. 1e). According to the PL decay curves (Fig. 1f) and the list of decay components (Table S1†), the PL lifetimes were significantly increased with an increase in the OA/OLA ratio. Thereby, the increase in the PL QY (from 11.5% to 35.4%) was found to derive from the decrease in the number or efficiency of the surface-related defect states. In the powder XRD patterns (Fig. 1g), all samples exhibit diffraction peaks at similar locations, which are assignable to the alloy composed of tetragonal AgInS_2_ and AgGaS_2_, indicating that the solvent composition does not significantly affect the crystalline component.

The aforementioned results indicated that the variation in the emission wavelength caused by changing the OA/OLA ratio seems to derive from the surface conditions. Interestingly, this idea corresponds to our previous finding that the broadband emission of the AIGS QDs originated from surface defects rather than from lattice defects.^23^ The fact that the contents of In, Ga, and S are higher than those at stoichiometry [Ag(In_*x*_Ga_1−*x*_)S_2_ (0 < *x* < 1)] has been commonly observed in the three samples, and it was revealed in our recent report that the amorphous indium and gallium sulfides that were deposited on the AIGS cores during synthesis affected the compositional analyses (Fig. S3†).^23^ However, the deviation from stoichiometry was slightly reduced when the AIGS QDs were synthesized in the mixture solvents. These results support the idea that OA, which is a strong X-type ligand, is useful to passivate the core QDs more strongly than OLA and to prevent the unnecessary deposition of byproducts.

**Fig. 2:**
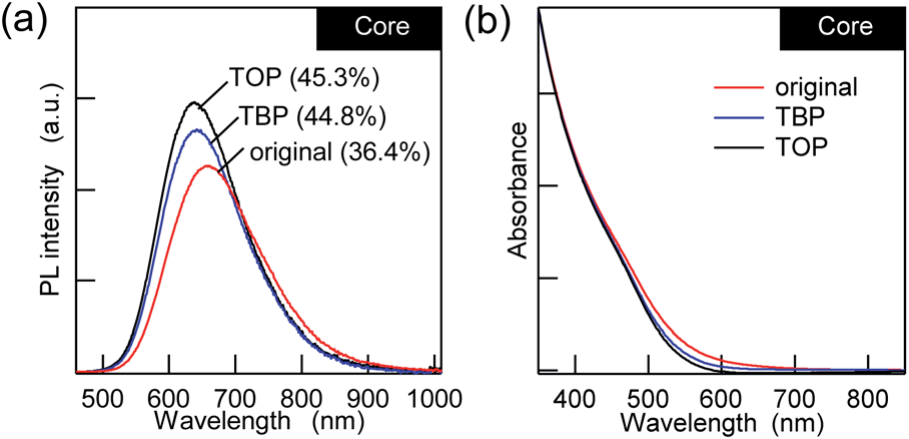
(a) PL and (b) UV-vis absorption spectra of the AIGS QDs with L-type ligands (TBP and TOP) in comparison with the original QDs.

Regarding the post-surface passivation, we have previously reported an increase in the PL QY for AgInS_2_/GaS_*y*_ by more than a factor of two^21^ and the remarkable recovery in the band-edge PL for the AIGS/GaS_*y*_^23^ core/shell QDs, which were achieved just by adding alkylphosphines. However, when TBP or TOP was added to the AIGS core QDs that were synthesized in the mixture solvent (OLA : OA = 2 : 1), the increase in the PL QY was only 10–20% (Fig. 2a), and it was accompanied by minor blue shifts of the PL. Since the UV-vis absorption spectra showed a decrease in the absorption tail that was present in the original AIGS QDs, the small changes in the PL can be explained in terms of the removal of the defect levels by phosphines. From the viewpoint of maintaining charge neutrality, there are no limitations for the binding of L-type ligands and ligand exchange with other L-type ligands. Therefore, phosphines should bind to the unpassivated metal sites and/or replace the existing OLA if the QDs possess these binding sites.^43^ The lower sensitivity of the PL to phosphine indicates that the AIGS QDs synthesized using Ga(DDTC)_3_ exposed sulfur sites more than metal sites, therefore being different from the previous AgInS_2_ core QDs (QY > 60%) that were synthesized from the thiourea derivatives.

### Z-type ligand coordination on the QD surfaces

**Fig. 3:**
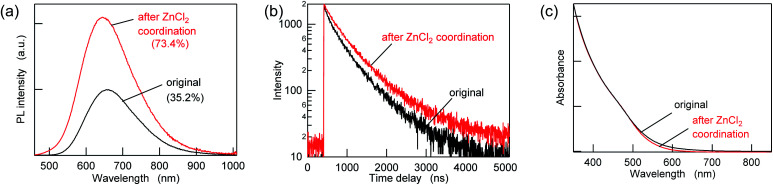
(a) PL spectra, (b) PL decay curves, and (c) UV-vis absorption spectra of the AIGS QDs before (original) and after ZnCl_2_ addition at a mole ratio of 10 000 ligands/QD.

To passivate the possible sulfur sites, Z-type ligands were mixed with the purified AIGS QDs dispersed in hexane. When 10 000 equivalents of ZnCl_2_ (compared to the amount of QDs in terms of nanoparticles) was added, followed by overnight incubation, an increase in the PL intensity of more than a factor of two was observed concomitantly with an increase in the PL lifetimes (Fig. 3a, b and Table S2†). A minor decrease in the UV-vis absorption was also observed in the region of 500–700 nm, suggesting the occurrence of surface passivation (Fig. 3c). The passivation seemed to be related to the binding of ZnCl_2_, since zinc and chlorine were detected from the treated AIGS QDs, as described in the following. The small amount of white precipitate that was found after completion of the reaction consisted of zinc and chlorine (Zn : Cl = 2 : 1) together with organic components. The ^1^H NMR spectrum of the precipitate measured in THF-*d*_8_ showed several peaks that were assignable to oleate, whereas other peaks were assumed to be the mixture of decomposed DDTC (Fig. S4†). The obtained results reveal that ZnCl_2_ not only binds to the dangling sulfur site but also provides the chloride ions that eventually remove the original ligands through the ligand exchange between X-type ligands.

**Fig. 4:**
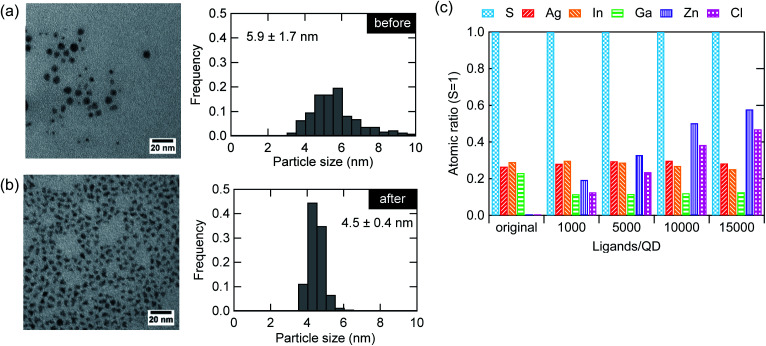
TEM images and corresponding size distribution histogram of the AIGS QDs (a) before and (b) after ZnCl_2_ addition at mole ratio of 10 000 ligands/QD. (c) Atomic ratio (sulfur basis) of the original AIGS QDs and the QDs after ZnCl_2_ addition at mole ratios in the range of 1000–15 000 ligands/QD.

In addition to the improvement in the optical properties of the AIGS QDs, the ZnCl_2_ treatment remarkably changed the particle morphology. Fig. 4a and b shows the TEM images before and after the treatment. The shape of the particles changed from spherical to angular together with a decrease in the particle size and size distribution. The change in the shape is due to the replacement of the surface ligands, which results in a variation in the surface energy, leading to changes in the facet of the lowest energy. Although the decrease in the average diameter was unexpected, a close look at the histogram indicates that such a decrease derives from the disappearance of larger particles (>5.5 nm). We speculate that the deposit deriving from the reactive DDTC complexes was effectively removed by the treatment, as has been discussed in a previous study as the cause of nonstoichiometry (excess of elements from groups 13 and 16).^23^Fig. 4c shows the elemental composition of the AIGS QDs before and after the treatment with different molar amounts of ZnCl_2_ to the QDs (1000–15 000). The ratios between the elements were displayed with respect to sulfur. A significant decrease in the ratios of indium, gallium, and sulfur was observed after the treatment with ZnCl_2_. The zinc and chlorine content increased steadily although these treated QD samples were purified carefully. The deviation of the Zn : Cl ratio from stoichiometry (1 : 2) might be caused by the hydrolysis of ZnCl_2_ due to exposure to moisture.

**Fig. 5:**
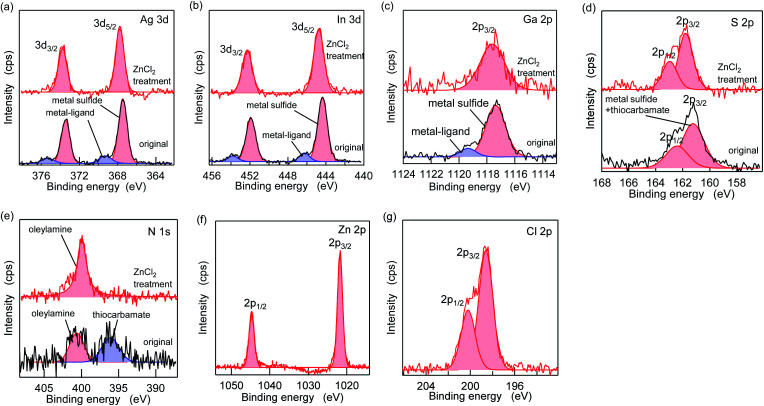
XPS spectra of the pristine AIGS QDs (black lines) and the ZnCl_2_-treated AIGS QDs (red lines). (a) Ag 3d, (b) In 3d, (c) Ga 2p, (d) S 2p, (e) N 1s, (f) Zn 2p, and (g) Cl 2p. (f) and (g) display the treated samples only.

**Scheme 1:**
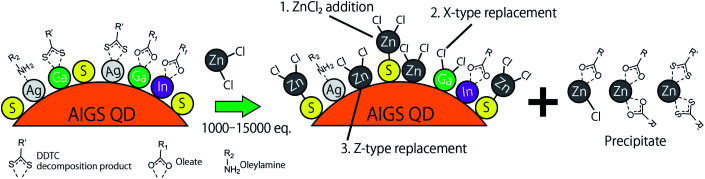
Schematic of the proposed mechanism of ligand exchange; (process 1) the addition of ZnCl_2_, (process 2) replacement of the X-type ligands, and (process 3) replacement of the Z-type ligands.

Fig. 5 shows the XPS spectra of the specific elements included in the AIGS QDs before and after the ZnCl_2_ treatment. As for Ag 3d, In 3d, and Ga 2p (Fig. 5a–c), the original AIGS QDs exhibited major peaks that are assignable to the metal sulfide as well as minor peaks located at higher binding energies. Since the elements of the minor peaks are more cationic than the metal sulfides, they are assignable to metal complexes bound on the surface of the QDs in a manner similar to the Z-type ligands. The ligands of these complexes are considered to be a mixture of oleate and the decomposition products of DDTC (Scheme 1, left). The disappearance of these minor peaks after the treatment with ZnCl_2_ supports the validity of these speculations. Furthermore, the S 2p peaks did not show any noticeable change after the treatment, indicating that the sulfur sites in the QDs are irrelevant to the reactions (Fig. 5d). Remarkably, one of the two N 1s peaks completely disappeared after the treatment (Fig. 5e). Since the L-type ligand (OLA) is mostly unaffected by the addition of Z-type ligands, the lower energy peak (396 eV) could be assigned to the decomposition product of DDTC. As expected from the composition analyses, Zn and Cl were detected clearly after the treatment (Fig. 5f and g). Based on the aforementioned results, we propose the three different processes during the ZnCl_2_ treatment that caused a significant improvement in the PL QYs (Scheme 1). The first is the bonding of ZnCl_2_ (Z-type ligand) to the sulfur sites, which was validated by the increase in the zinc and chlorine content after the treatment (process 1). Therefore, the majority of the increase in the PL QY is due to the passivation of the dangling sulfur sites.^28^ Another possible reaction is a simple exchange of the X-type ligands with the chloride ions of ZnCl_2_ (process 2), which is supported by the presence of the white precipitate consisting of zinc, oleate, and the decomposition products of the DDTC ligands.^37,44^ In addition, the decrease in the ratio of the original metal species (specifically of gallium) indicates the desorption of these metals by means of the ligand exchange with ZnCl_2_ (process 3).

Fig. S5† summarizes the increase in the PL QY and average lifetimes after treatment with the different amounts of ZnCl_2_ for the AIGS QDs synthesized in the different mixture solvents (OLA only, OLA : OA = 2 : 1, and OLA : OA = 1 : 2). Except for the OLA-only case, the PL QY and lifetime varied independently as a function of the ZnCl_2_ amount. These results indicate that not only the passivation of nonradiative trap states but also changes in the emission mechanisms occurred due to the ZnCl_2_ treatment. In addition, the saturation of the PL QY in the presence of a high amount of ZnCl_2_ (>10 000 ligands/QD) is due to the saturation of the bindable sites, and it also causes a decrease in the dispersibility in a nonpolar environment. Interestingly, when other metal chlorides and oleates were added to the AIGS QDs in the same manner as ZnCl_2_ (10 000 ligands/QD), the increase in the PL QY was lower than that in ZnCl_2_. The PL intensity variations caused by the treatment were found to be 125% (absolute PL QY value increased from 35.2% to 44.1%) for zinc oleate (Zn[OA]_2_) and 136% (from 31.8% to 43.4%) for indium chloride (InCl_3_), whereas the PL QY value decreased to 86% (from 37.0% to 31.9%) when gallium chloride (GaCl_3_) was added. All these values are much lower than that for ZnCl_2_ (change ratio was 208%, PL QY value increased from 35.2% to 73.4%). These results indicate that the increment in the PL QY depends strongly on the type of Z-type ligands. However, this trend in the PL QY values does not appear to follow the simple trend of the Lewis acidity, since GaCl_3_, which is a stronger Lewis acid than InCl_3_, resulted in a decrease in the PL QY. This circumstance (*i.e.*, the mismatch between the PL QY and the Lewis acidity) is similar to that of a recent report, in which the variations in the PL QY of core-only cadmium telluride (CdTe) QDs were investigated under post-treatment with various types of ligands. Although the Z-type ligands increased the PL QY considerably more than other types of ligands, the order of the increment does not always follow the binding strength of the ligands.^28^ Even a steric hindrance was reported to affect the binding of the ligands and the increment in the PL QY, which is consistent with our results that Zn(OA)_2_ is less effective in improving the PL QY than ZnCl_2_.

### Z-type ligand passivation on the band-edge emitting QD

As mentioned in the introductory remarks, we previously found that the coating of AgInS_2_ (ref. 21 and 45) and AIGS^23^ QDs with GaS_*y*_ produced core/shell QDs exhibiting a spectrally narrow band-edge emission. Recently, we obtained a similar type of emission from AIGS QDs synthesized in one batch by heating the raw material solution at a temperature higher than 260 °C. The successful observation of the band-edge emission *via* the one-batch reaction was considered to be due to the decomposition of highly reactive Ga(DDTC)_3_ by itself to generate the GaS_*y*_ shell precursor after the Ag and In sources were depleted. The advantage of the one-batch reaction in synthesizing core and core/shell QDs continuously is the simplicity of the procedure as well as the reduction in the damages that are inevitably caused to the core during isolation. However, the PL QY of the core/shell-like QDs obtained *via* the one-batch reaction was lower (∼9%) (Fig. S6†) than that of the core/shell sample obtained *via* the separate process using gallium acetylacetonate and 1,3-dimethylthiourea.^23^

**Fig. 6:**
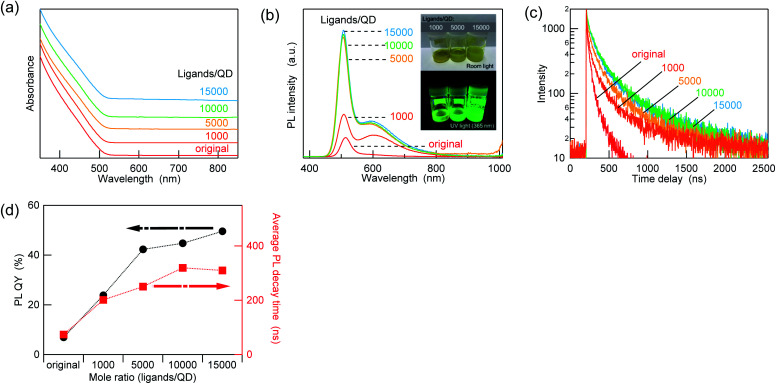
(a) UV-vis absorption spectra, (b) PL spectra, and (c) PL decay curves of the AIGS/GaS_*y*_ core/shell-like QDs before and after the treatment with ZnCl_2_ at different molar ratios (1000–15 000 ligands/QD). The UV-vis absorption spectra are offset for better clarity, and the insets in (b) shows photographs of the samples under ambient light and UV light. The PL decay curves were recorded at an excitation wavelength of 365 nm and an observation wavelength of 507 nm. (d) Variations in the PL QYs and average lifetimes as a function of the ZnCl_2_ amount.

Fig. 6a and b shows the UV-vis absorption and PL spectra of the core/shell-like QDs before and after the ZnCl_2_ treatment. Interestingly, the treatment with the Z-type ligand increased the PL intensity of both the band-edge and remaining defect emissions, whereas the absorption profile was almost unchanged. Such changes in the optical properties are similar to those observed for the AIGS core QDs. The consecutive extension of the PL decay time was observed upon increasing the ZnCl_2_ amount (Fig. 6c). The variation in the average lifetimes is in good agreement with the PL QY, and an approximately fivefold increase in the PL QY (49.5%) was achieved after the treatment (Fig. 6d). Similar to the AIGS cores, the XPS spectra of the core/shell-like QDs showed the presence of Zn and Cl after the treatment, indicating the coordination of ZnCl_2_ on the surface sites of the QDs (Fig. S7†). Although minor shifts in the binding energy were commonly observed for the Ag, In, Ga, and S peaks of the QDs after the treatment, the offsets were the same for all data. Therefore, the small high-energy shifts might be due to changes in the charging state of the QD body caused by the ligand exchange, as expected from the fact that this data was taken using the C 1s peak as an energy standard.

## Conclusions

We have demonstrated the improvement in the PL efficiency of quaternary AIGS QDs by changing surface ligands. The use of the OA/OLA mixed solvent instead of pure OLA increased the PL QY of the AIGS QDs from 11.5% to 35.2%, which is considered to be due to the fact that the X-type OA can passivate multicomponent QDs more strongly than can the L-type OLA. On the other hand, the color of the emission of the AIGS QDs changed from yellow to red upon increasing the OA proportion in the mixed solvent, indicating that the observed broadband PL is derived from the surface. The higher binding strength of the X-type OA was supported by the decrease in the particle size and size distribution when the OA content was increased. The post-synthetic modifications were further investigated using AIGS QDs prepared with the mixture solvent. While the addition of alkylphosphines (L-type ligands) increased the PL QY by only 20%, the PL QY value doubled (from 35.2% to 73.4%) when ZnCl_2_ (Z-type ligand) was mixed with the AIGS QD solution. Since Z-type ligands bind specifically to sulfur sites, the AIGS QDs prepared using a highly active sulfur source (Ga[DDTC]_3_) had many sulfur sites on their surface, which was revealed to be a main reason for the low PL QY.

The ZnCl_2_ treatment was further applied to the AIGS/GaS_*y*_ core/shell-like QDs that were synthesized in one batch and had a band-edge emission. A fivefold increase in the PL QY (from 9% to 49.5%) was achieved after treatment with the Z-type ligands, and the intensity of the defect emission increased concomitantly. Based on the analyses of the treated QDs and byproducts, three possible mechanisms were proposed concerning the addition of the Z-type ligands, namely, the bonding of ZnCl_2_ to the sulfur sites, the replacement of the X-type ligands between the QDs and ZnCl_2_, and the replacement of the Z-type ligands themselves. We expect that the deeper understanding of the ligands gained through these results will improve the performance and usability of the multinary QDs.

## Author contributions

WH – conceptualization, synthesis of materials, analyses, and writing original draft; TU – visualization, analyses, review & editing, and funding acquisition; TT – supervision; SK – supervision, funding acquisition, and review & editing.

## Conflicts of interest

There are no conflicts to declare.

## Supplementary Material

NA-004-D1NA00684C-s001
