# Supplementary material for: Surface ligand chemistry on quaternary Ag(InxGa1−x)S2 semiconductor quantum dots for improving photoluminescence properties
Source: Nanoscale Adv. 2021 Dec 10;4(3):849–57. doi: 10.1039/d1na00684c (PMC9419514; doi:10.1039/d1na00684c)
Supplement: NA-004-D1NA00684C-s001 [file NA-004-D1NA00684C-s001.pdf]

Electronic Supplementary Material (ESI) for Nanoscale Advances.
This journal is © The Royal Society of Chemistry 2022

Electric Supplementary Information

Surface Ligand Chemistry on Quaternary $Ag(In_xGa_{1-x})S_2$ Semiconductor Quantum Dots for Improving Photoluminescence Properties

Authors: Watcharaporn Hoisang,[1] Taro Uematsu,[1,2*] Tsukasa Torimoto,[3] and Susumu Kuwabata[1,2*]

Affiliations:

[1] Department of Applied Chemistry, Graduate School of Engineering, Osaka University, 2-1 Yamada-oka, Suita, Osaka 565-0871, Japan

[2] Innovative Catalysis Science Division, Institute for Open and Transdisciplinary Research Initiatives (ICS-OTRI), Graduate School of Engineering, Osaka University, 2-1 Yamada-oka, Suita, Osaka 565-0871, Japan

[3] Department of Materials Chemistry, Graduate School of Engineering, Nagoya University, Chikusa-ku, Nagoya 464-8603, Japan

Correspondence: Taro Uematsu (t-uematsu@chem.eng.osaka-u.ac.jp) and Susumu Kuwabata (kuwabata@chem.eng.osaka-u.ac.jp)

Synthesis of gallium diethydithiocarbamate

Gallium diethyldithiocarbamate [Ga(DDTC)$_3$] was prepared through a method taken from the literature, which was subsequently modified.[1] A 50 mL volume of an aqueous solution of 0.3 M NaDDTC was added dropwise into 50 mL of an aqueous solution of 0.1 M Ga(NO$_3$)$_3$. A white precipitate was formed immediately. The solution was stirred at room temperature for 2 h. The product obtained was filtered, washed with deionized water, and dried in a vacuum oven at room temperature overnight.

Synthesis of zinc oleate

Zinc oleate (Zn[OA]$_2$) was synthesized using the following procedure.[2] ZnO (1.66 g) was mixed with excess oleic acid (13.6 g) in a two-neck round-bottomed flask. The solution was subsequently degassed and heated to 260°C in an argon atmosphere. The solution was cooled down to ambient temperature; the product was then purified with toluene/acetone. The white solid Zn(OA)$_2$ was finally dried and kept at room temperature.

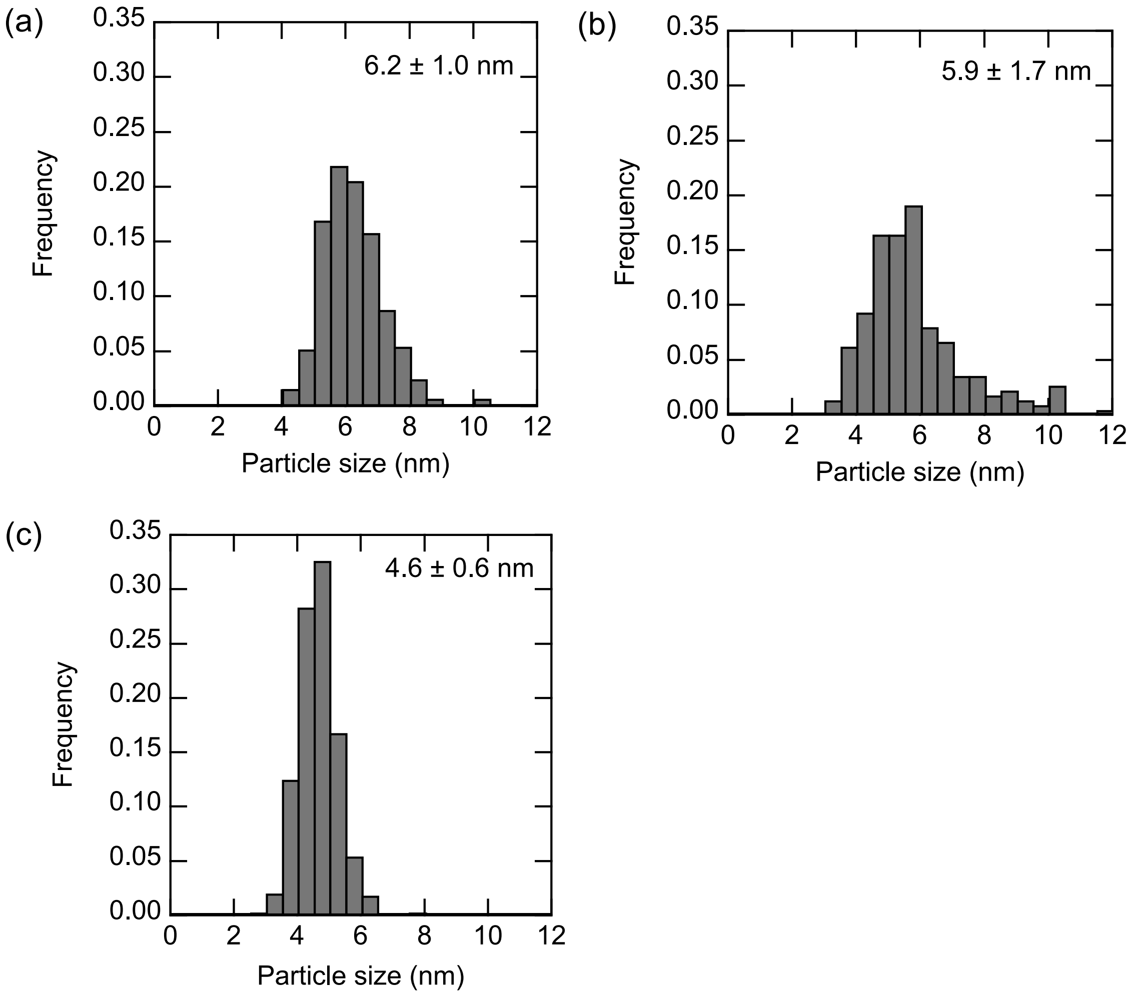

Fig. S1 Corresponding size distributions of Fig. 1(a–c) for the as-synthesized AIGS QDs in different solvents: (a) OLA, (b) OLA:OA = 2:1, and (c) OLA:OA = 1:2.

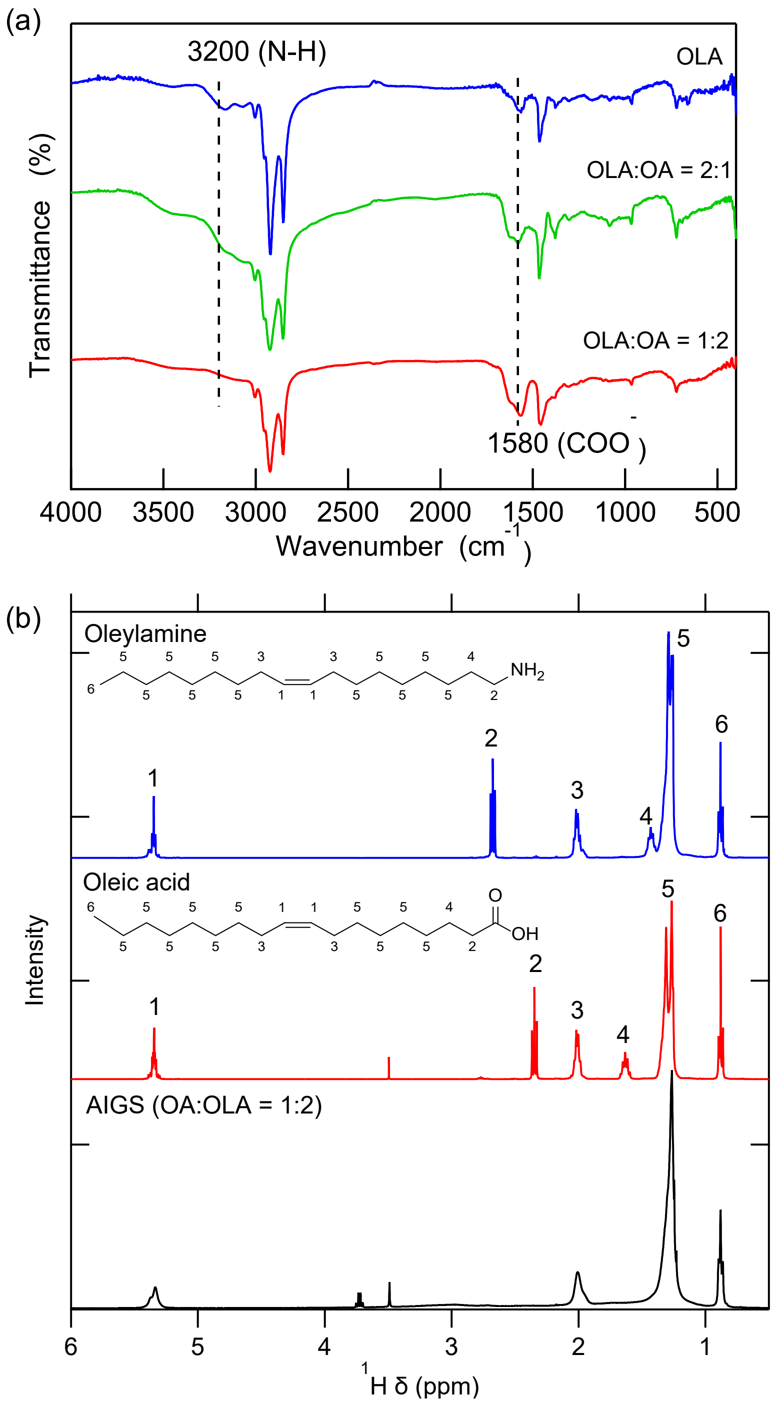

Fig. S2 (a) FT-IR spectra of the AIGS QDs synthesized with OLA only and an OLA:OA mixture (with ratios 2:1 and 1:2). (b) ^{1}H NMR spectra (chloroform-d) of free OLA and OA as well as of AIGS QDs synthesized in an OLA:OA mixture solvent (2:1).

Equation 1 The multiexponential function for the PL decay calculation is

$$I(t) = \sum_i A_i \exp\left(-\frac{t}{\tau_i}\right), \quad (1)$$

where $I(t)$, A_i, and τ_i are the decay curve, amplitude, and lifetime, respectively. This equation is used for calculating the data in Table S1 and S2.

Table S1 Fitted PL lifetime components derived from the triexponential function ($I(t) = A_1 \exp(-t/\tau_1) + A_2 \exp(-t/\tau_2) + A_3 \exp(-t/\tau_3)$) of the AIGS QDs corresponding to Fig. 1f.

AIGS	$\langle\tau_{av}\rangle$ (ns)	τ_1 (ns)	A_1	τ_2 (ns)	A_2	τ_3 (ns)	A_3	χ^2
OLA	262.6	23.9	0.41	144.8	0.43	405.4	0.17	1.04
OLA:OA = 2:1	603.4	33.9	0.18	249.1	0.41	734.2	0.41	1.02
OLA:OA = 1:2	1042.8	72.2	0.18	512.1	0.57	1487.8	0.25	1.06

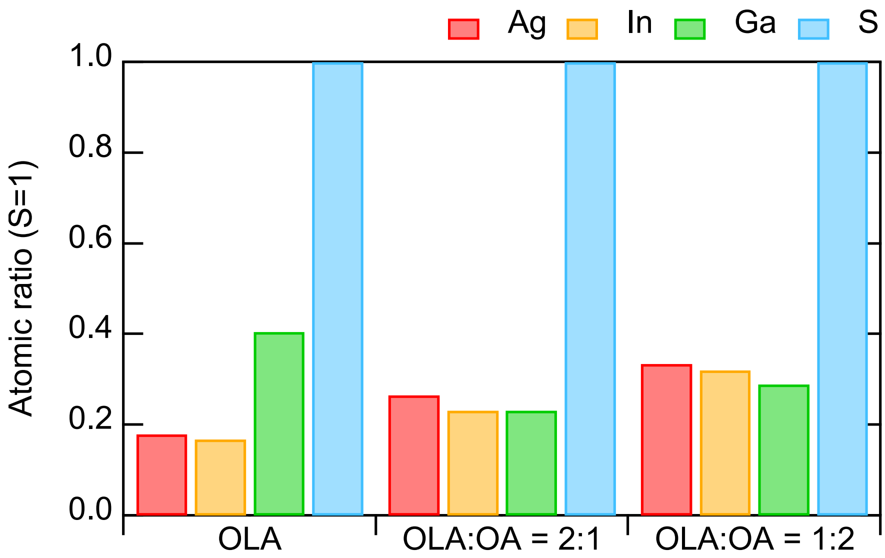

Fig. S3 Atomic ratios (sulfur basis) obtained via the EDX–SEM analysis for the AIGS QDs synthesized with various surface ligands.

Table S2 Fitted PL lifetime components of the AIGS QDs corresponding to Fig. 3b.

AIGS	$<\tau_{av}>$ (ns)	τ_1 (ns)	A_1	τ_2 (ns)	A_2	τ_3 (ns)	A_3	χ^2
Before treatment	603.4	33.9	0.18	249.1	0.41	734.2	0.41	1.02
After treatment	691.7	34.1	0.17	328.3	0.50	903.8	0.33	1.02

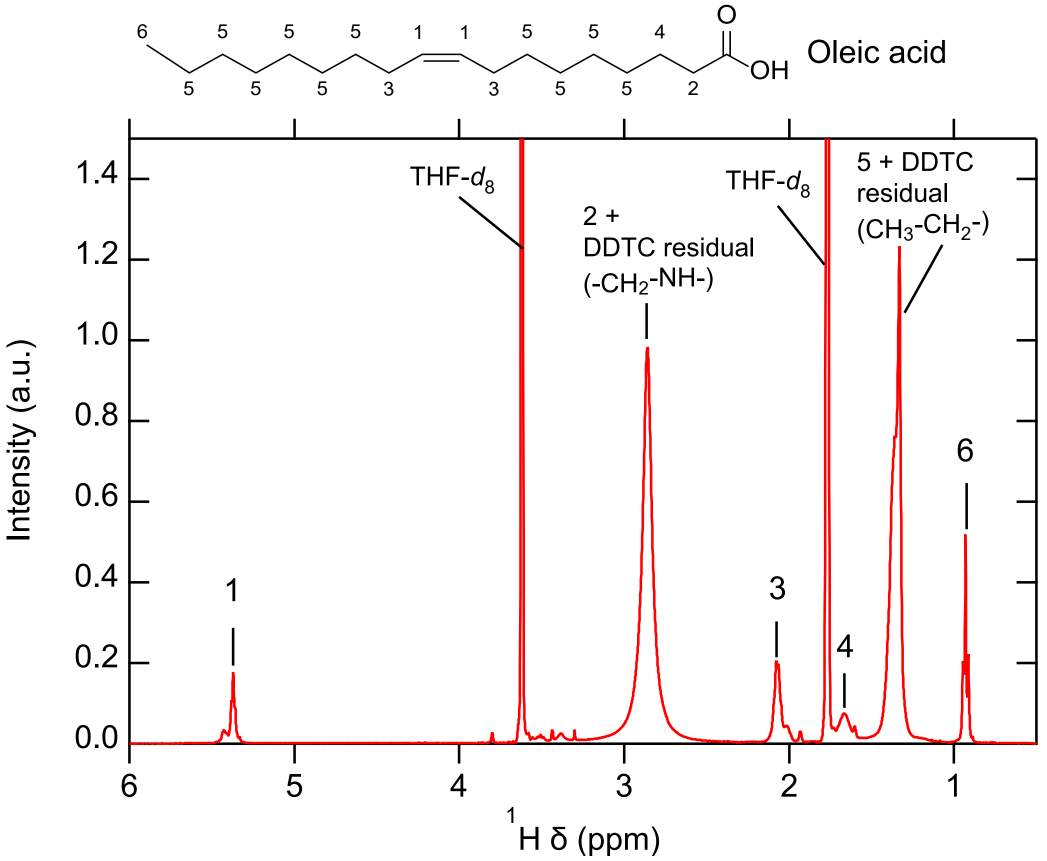

Fig. S4 (a) ^{1}H NMR spectra (THF-d_8) of the white precipitate that was generated after the $ZnCl_2$ treatment of the AIGS QDs synthesized in the OLA:OA mixture solvent (with ratio 2:1).

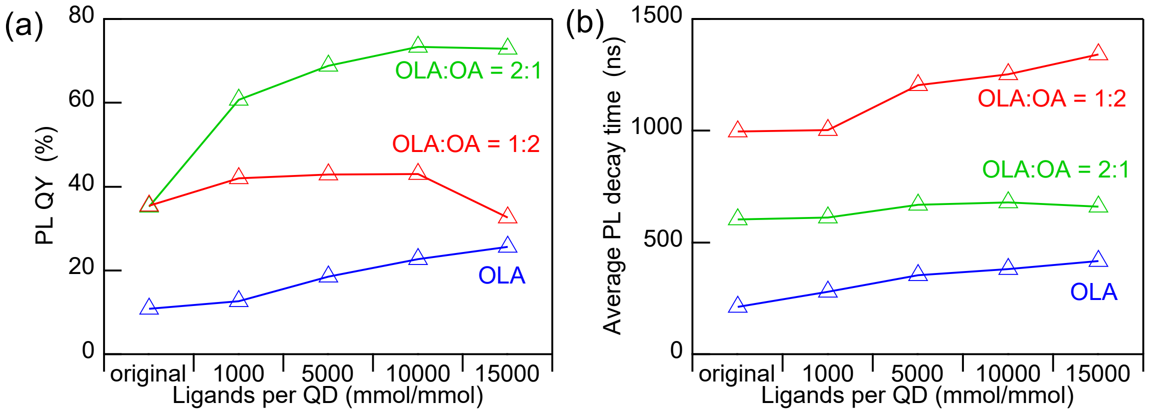

Fig. S5 Change in (a) the PL QY values and (b) the average PL decay times of the AIGS QDs synthesized using various ligands (OLA, OLA:OA = 2:1, and OLA:OA = 1:2) before and after the $ZnCl_2$ treatment at 1,000–15,000 ligands/QD.

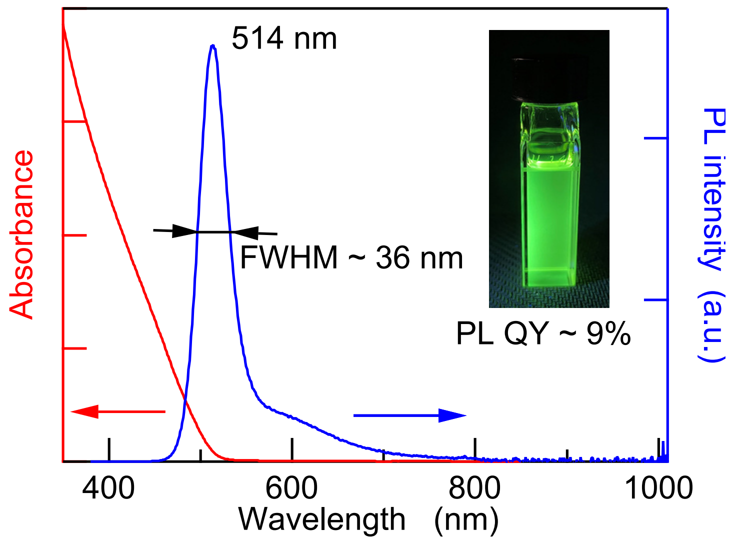

Fig. S6 UV–vis absorption and PL spectra of the AIGS/GaS$_y$ core/shell-like QDs with the inset photograph showing the QD solution under UV radiation (365 nm); the photographed QD solution has a PL QY of approximately 9%, as labeled in the figure.

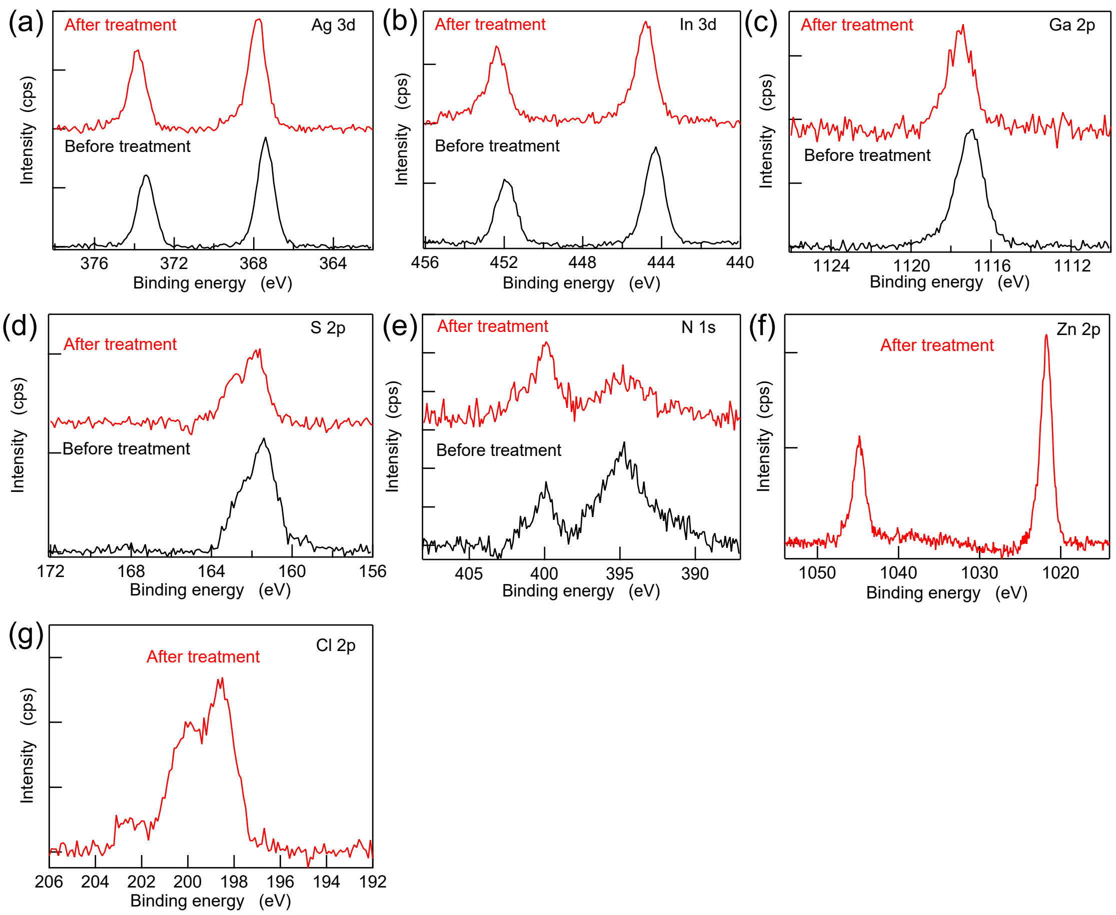

Fig. S7 XPS spectra of the AIGS/GaS_y core/shell-like QDs before (black curves) and after (red curves) the $ZnCl_2$ treatment: (a) Ag 3d, (b) In 3d, (c) Ga 2p, (d) S 2p, (e) N 1s, (f) Zn 2p, and (g) Cl 2p.

References

1. T. X. Wang, H. Xiao and Y. C. Zhang, *Mater. Lett.*, 2008, **62**, 3736-3738.
2. T. Yao, Q. Zhao, Z. Qiao, F. Peng, H. Wang, H. Yu, C. Chi and J. Yang, *Chemistry*, 2011, **17**, 8663-8670.
